# The effect of citalopram treatment on amyloid-β precursor protein processing and oxidative stress in human hNSC-derived neurons

**DOI:** 10.1038/s41398-022-02050-5

**Published:** 2022-07-18

**Authors:** R. J. Elsworthy, J. A. Crowe, M. C. King, C. Dunleavy, E. Fisher, A. Ludlam, H. R. Parri, E. J. Hill, S. Aldred

**Affiliations:** 1 School of Sport, Exercise and Rehabilitation Sciences, College of Life and Environmental Sciences, University of Birmingham, Birmingham, UK; 2 Centre for Human Brain Health (CHBH), University of Birmingham, Edgbaston, Birmingham, UK; 3 School of Biosciences, College of Health and Life Sciences, Aston University, Birmingham, UK; 4 Department of Clinical Sciences, Division of Neurology, Lund Stem Cell Centre, Faculty of Medicine, Lund University, Lund, Sweden; 5 Department of Physiology and Biophysics, State University of New York at Buffalo, Buffalo, NY USA

**Keywords:** Molecular neuroscience, Stem cells

## Abstract

Selective Serotonin Reuptake Inhibitors (SSRIs) may hold therapeutic benefits for people with Alzheimer’s disease (AD). SSRIs may perturb AD progression, or the conversion from MCI to AD, via increased neurogenesis, reduced oxidative stress and/or favourable Amyloid-β Precursor Protein (AβPP) processing. This study used iPSC derived cortical neuronal cells carrying 3 different PSEN1 mutations, to investigate the effect of treatment with the SSRI, Citalopram on AβPP processing and oxidative stress. Control and PSEN1 mutation (L286V, A246E, M146L) iPSC-derived neurons were treated with Citalopram for 45 days. ADAM10 activity, AβPP processing and Aβ generation was measured in addition to cellular redox status. Citalopram treatment reduced the Aβ1-42:40 ratio in control but not in fAD PSEN1 cells. ADAM10 activity was increased with Citalopram treatments in fAD PSEN1 cell lines, which was also seen for sAβPPα secretion. Lower superoxide generation in fAD PSEN1 cells following Citalopram treatment was identified, although there was no effect on end markers of oxidative stress. Treatment with Citalopram appears to have little effect on Aβ generation in fADPSEN1 cells, but our findings suggest that treatment can significantly increase non-amyloidogenic AβPP processing and reduce oxidative stress. These changes may explain why SSRIs appear most effective in the prodromal period of the disease progression, as opposed to reducing established AD pathology. Further investigation of specific pathways conferring the beneficial effects of SSRIs treatment are warranted.

## Introduction

Selective serotonin reuptake inhibitors (SSRIs) target serotonin receptor transporters (SERT), preventing serotonin re-uptake from the synapse. The use of SSRIs may modify some disease process via increased neurogenesis, reduced oxidative stress and favourable Amyloid-β Precursor Protein (AβPP) processing [1], which has generated interest in their ability to treat Alzheimer’s disease (AD).

When sequentially cleaved by beta-site amyloid precursor protein cleaving enzyme-1 (BACE-1) and γ-secretase, AβPP is broken down into sAβPPβ, β C-terminal fragment and the Aβ peptide. The aggregation of Aβ into soluble oligomers and Aβ plaques are hallmark features of the AD brain and thought to contribute to the disease progression [2]. Alternatively, AβPP may be cleaved via α-cleavage, attributed chiefly to ‘a disintegrin and metalloproteinase’ 10 (ADAM10) followed by γ-secretase [3]. Cleavage of AβPP by ADAM10 precludes Aβ generation, and releases a neuroprotective s*A*βPP-α n-terminal fragment and an α C-terminal fragment [4]. Thus, this direction of *A*βPP processing is typically considered favourable in preventing AD related amyloid pathology.

There is evidence that modulation of serotonin signalling alters non-amyloidogenic AβPP processing [5, 6]. Recent studies have focussed on modulation of the serotonin receptors (5HTr), 5HT4r and 5HT6r, as they can regulate AβPP [7]. Understanding the effects of serotonin modulation via SSRIs on AβPP processing is crucial for guidance to be given on the use of SSRIs to prevent or delay the progression of AD.

Previous studies have shown that neurons derived from hNPCs carrying a PSEN1 familial AD mutation display altered AβPP processing [8, 9]. Mutations in PSEN1, which code for the PSEN1 subunit of γ-secretase, may reduce enzymatic function and the maturation of γ-secretase, which can lead to altered carboxypeptidase-like activity [8]. Thus, hNPCs are a suitable platform to investigate mechanisms of the pharmacological action of SSRIs as they appear to reflect early pathological features associated with AD. The aim of this study was to investigate how chronic Citalopram treatment of hNPC-derived neurons carrying PSEN1 mutations (L286V, A246E, M146L) affected AβPP processing and redox balance.

## Materials and methods

All reagents were purchased Sigma-Aldrich (St Louis, MA) unless otherwise stated. Cells were routinely tested for Mycoplasma contamination by luminescence assay (Lonza MycoAlert^TM^ PLUS, LT07-703). For information on cell lines used see Table 1.

**Table 1:** Information on cell lines used for healthy control and for modelling AD phenotype.

	Ax0018	Ax0019	Ax0112	Ax0113	Ax0114
Diagnosis	Healthy Control	Healthy Control	Familial AD	Familial AD	Familial AD
Sample type	Dermal Fibroblast	Dermal Fibroblast	Dermal Fibroblast	Dermal Fibroblast	Dermal Fibroblast
Donor sex	Male	Female	Female	Female	Male
Age at sampling (yrs)	74	64	38	31	53
Age of onset (yrs)	n/a	n/a	39	45	n/a
Karyotype	Normal	Normal	Normal		
Reprogramming method	Episomal Vector	Episomal Vector	Episomal Vector	Episomal Vector	Episomal Vector
Induction method	Monolayer – Axolbio NIM (Shi et al., 2012)	Monolayer – Axolbio NIM (Shi et al., 2012)	Monolayer – Axolbio NIM (Shi et al., 2012)	Monolayer – Axolbio NIM (Shi et al., 2012)	Monolayer – Axolbio NIM (Shi et al., 2012)
Mutation	None	None	PSEN1 (L286V)	PSEN1 (A246E)	PSEN1 (M146L)
APOE status	ε2/ ε2	ε3/ ε3	ε3/ ε3	ε2/ ε3	ε3/ ε4

Adapted from [9].

### Expansion of human neural stem cells

Healthy control (ax0018, ax0019) and PSEN1 mutations carrying L286V (ax0112), A246E (ax0114) and M146L (ax0113) hNPCs were purchased from Axol Bioscience (Cambridge, UK) and cultured as previously described [9]. Briefly, Control and fAD PSEN1 cells were seeded at a density of 7 × 10^4^ cells/cm^2^ in neural maintenance media (NMM, Axol Bioscience, Cambridge, UK) on poly-L-ornithine (20 μg/mL, A-004-M) and murine laminin from EHS (10 μg/mL, L2020) coated wells. Cells were passaged at 80% confluency using 1 mL/well Accutase™ (A6964, Merck, UK) and dissociation stopped with 4 mL/well NMM. Cells were maintained in a 37 °C, 5% CO_2_/95% air atmosphere and total media exchanged every other day. Each replicate (n) was expanded through 2 passages, for final plating at passage 3. Each cell line was analysed in a minimum *n* = 3.

### Citalopram treatments

Citalopram Hydrobromide (10 mg, C7861) was initially prepared in methanol, followed by ultrapure sterile filtered dH_2_O (5000 μM) before storage at −20 °C. Treatment with Citalopram began 24 h after final passage and was delivered in NMM with a full media exchange every 48 h. Citalopram was delivered at 0.8 μM, 5 μM and 10 μM concentrations [10, 11] based of MTT assay viability (*see MTT assay*) over 44 days giving a total culture period of 45 days. At day 45, cells were lysed in ice-cold lysis buffer (200 mM NaCl, 10 mM EDTA, 10 mM Na_2_HPO_4_, 0.5% NP40, 0.1% SDS, 1x protease inhibitors and 5 µM GI254023X, pH 8.0), and media was collected for storage (-80 °C) and subsequent analysis.

### Immunocytochemistry

Cells were fixed in 4% (v/v) paraformaldehyde (PFA) in D-PBS. The cells were then incubated for 10 min in PBS with 0.2% (v/v) Triton X-100 followed by blocking for 1 h in PBS containing 0.2% (v/v) Triton X-100 and 2% (w/v) bovine serum albumin (A9418). Primary antibodies for MAP2 (MA5-12826, Invitrogen), Synaptophysin (MA5-14532, Invitrogen), Serotonin reuptake transporters (SERT) (702076, Invitrogen), AβPP (Merck, Mab348) and ADAM10 (Abcam, AB1997) were diluted in blocking buffer and added for 1 h. Following primary antibody incubation, cells were washed with blocking buffer and appropriate secondary antibodies, Alexa Fluor® 488 AffiniPure Goat Anti-Rabbit IgG (1:2000, 111-545-144, Jackson Laboratories) and Alexa Fluor® 633 Goat anti-Mouse IgG (1:2000, A-21052, ThermoFisher Scientific) were added for 1 h. Cells were mounted in Prolong^TM^ Gold Antifade Mountant with DAPI (P3935, ThermoFisher Scientific) to glass slides and imaged using a Nikon A1R laser scanning confocal microscope (Nikon EU, Netherlands).

### Calcium imaging

Cells were incubated with the labelled calcium indicator Fluo-4AM (ThermoFisher Scientific, F14201) in culture medium for 45 min (10 µM, 37 °C). Cell were incubated for 5 min (37 °C) to allow for de-esterification of AM esters. For baseline measurements of calcium signalling, artificial CSF (aCSF) was perfused over the cells for 5 min (see Table 2). 5HT (10 µM in dH_2_O, 14927) was perfused for 5-minutes suspended in aCSF before a 5-min aCSF washout period. A minimum time-course of 3-min has been used in previous research to investigate 5HT induced calcium dynamics at similar compound concentrations [12, 13]. Imaging was performed using a fluorescence microscope (Nikon Eclipse FN1) with a 20× objective. Fluo-4AM fluorescence was excited at 488 nm and captured every 2 s to create a time-lapse (Hamamatsu Orca Flash V2). The fluorescence was calculated from five regions of interest containing approximately 3–10 cells using Fiji software (ImageJ, NIH).

**Table 2:** aCSF constituent used for perfusion over cells for baseline calcium measurements and as a vehicle for serotonin.

Component	Final concentration (mM)
NaCl	126
KCL	2.5
NaHCO_3_	26
KH_2_PO_4_	1.25
MgSO_4_	1
CaCl_2_	2
Glucose	10

pH 7.3–Continuous bubbling with CO_2_ throughout the experiment.

### MTT assay

hNPCs were seeded (1.5 × 10^5^/cm^2^) into 96 well plates as previously described. Triplicate repeats for each control and experimental condition were used. Treatments were serially diluted and delivered using NMM as the vehicle which was exchanged prior to the assay. Control wells were prepared in the vehicle alone. For the assay, 3-(4,5-dimethylthiazol-2-Yl)-Diphenyltetrazolium Bromide (MTT, CT01-5) stock solution was diluted in NMM (1:5), added to each well and incubated for 3 h (37 °C). The MTT solution was then aspirated and DMSO (50 μl) was added to each well. Cells were placed on a plate shaker (500 rpm) for 30 s followed by incubation for 10 min (37 °C). Finally, absorbance was read at 590 nm (Fluostar Omega, BMG Labtech). Percentage viability was calculated from control cells using a Log dose–response curve.

### Immunoassays

Immunoassays for Aβ1-40 (ThermoFisher, KHB3481), Aβ1-42 (ThermoFisher KHB3441), aggregated Aβ (ThermoFisher, KHB3491), and sAβPP-α (MyBioSource, MBS9358454) in conditioned media was measured via ELISA according to manufacturer’s instruction. Total 8-isoprostanes were measured via ELISA (516351, Cayman Chemical) in cell lysates following purification using the 8-isoprostane affinity sorbent (401113, Cayman Chemical) suspended in Eicosanoid Column Affinity buffer (400220, Cayman Chemical). Protein carbonylation was assessed by the method of Carty et al. (2000).

### ADAM10 activity

ADAM10 activity was measured via fluorometric FRET assay following manufacturer’s instructions (AS-72226, Sensolyte 520, Anaspec). Cells were seeded (1.5 × 10^5^/cm^2^) onto 96 well plates. Next, purified ADAM10 enzyme (positive control), assay buffer (negative control) and was added to appropriate wells. Standards (50 μL) were added to wells using the 5-FAM peptide (0–5 μM). Next, ADAM10 substrate solution (50 μL) was added to each well. The Plate was then incubated at 37 °C for 45 min and read at fluorescence intensity ex/em = 490 nm/520 nm (Fluostar Omega, BMG Labtech). Linear regression was used to calculate sample ADAM10 activity in relative fluorescence units (RFU) compared to 5-FAM peptide.

### MitoSOX^TM^ Red superoxide indicator

MitoSOX^TM^ Red mitochondrial superoxide indicator (M36008, Invitrogen) was applied to cells at a final concentration of 5 μM and incubated (37 °C) for 10 min. Fluorescence was captured using an Inverted microscope (CKX53SF, Olympus life science). Quantification of the fluorescence intensity that oxidized the MitoSOX^TM^ reagent was performed with Fiji (ImageJ, NIH) and averaged for three independent experiments.

### Statistical analysis

For experiments it was determined that a relatively small sample size was needed (*n* = 3, 3 replicates/experiment) for 90% power (1-beta) at 5% significance (alpha) based on prior data collection in similar cell models [9]. To generate a sigmoidal dose-response curve for Citalopram treatment or standard curves from plate-based assays either linear regression or 4-paramaeter logistic regression was used to plot known concentrations against optical absorbance at specified wavelengths. From this sample concentrations were calculated and normalized to total protein concentration in corresponding cell lysate. All quantitative data in the text and figures are presented as Mean ± SD unless otherwise stated. Normal distribution and SD variance were tested using Shapiro-Wilk and Brown-Forsythe testing respectively. Significance was calculated using ordinary one-way ANOVA with Bonferroni *post hoc* corrections. All data were processed using GraphPad Prism (Version 9.3.1).

## Results

### hNPC-derived neurons are a viable model to investigate the effects of Citalopram treatment on markers of AD phenotype

**Fig. 1:**
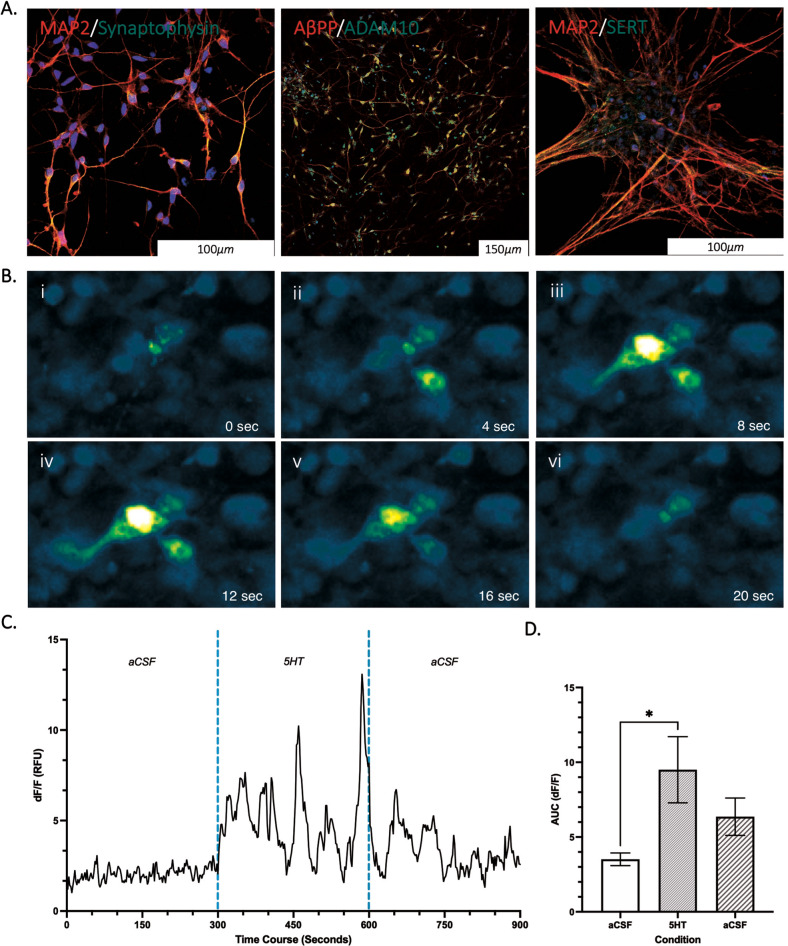
Representative microscopy images of cultured cells with ICC staining (A) and calcium imaging (B & C) (*n* = 3). **A** Mature neuronal staining positive for MAP2, Synaptophysin, SERT, ADAM10 and AβPP (AX0018, d45) with nucleus staining for DAPI (blue). **B** PSEN1 (L286V) fluoro-4-am calcium transient measured in neurons (day 42). **C** Signal intensity was used to calculate neuronal activity (DF/F) under basal (aCSF), during stimulation with 5HT and a wash off period (aCSF) in 5-min intervals (*n* = 3). **D** Area under the curve plotted for each condition to analyse neuronal activity (3ROIs, *n* = 3). Data normality (p > 0.05) and S.D. Variation (p < 0.05) confirmed assumptions for ANOVA analysis with multiple comparison corrections applied. *Significantly different to treatment condition (*p* < 0.05).

**Fig. 2:**
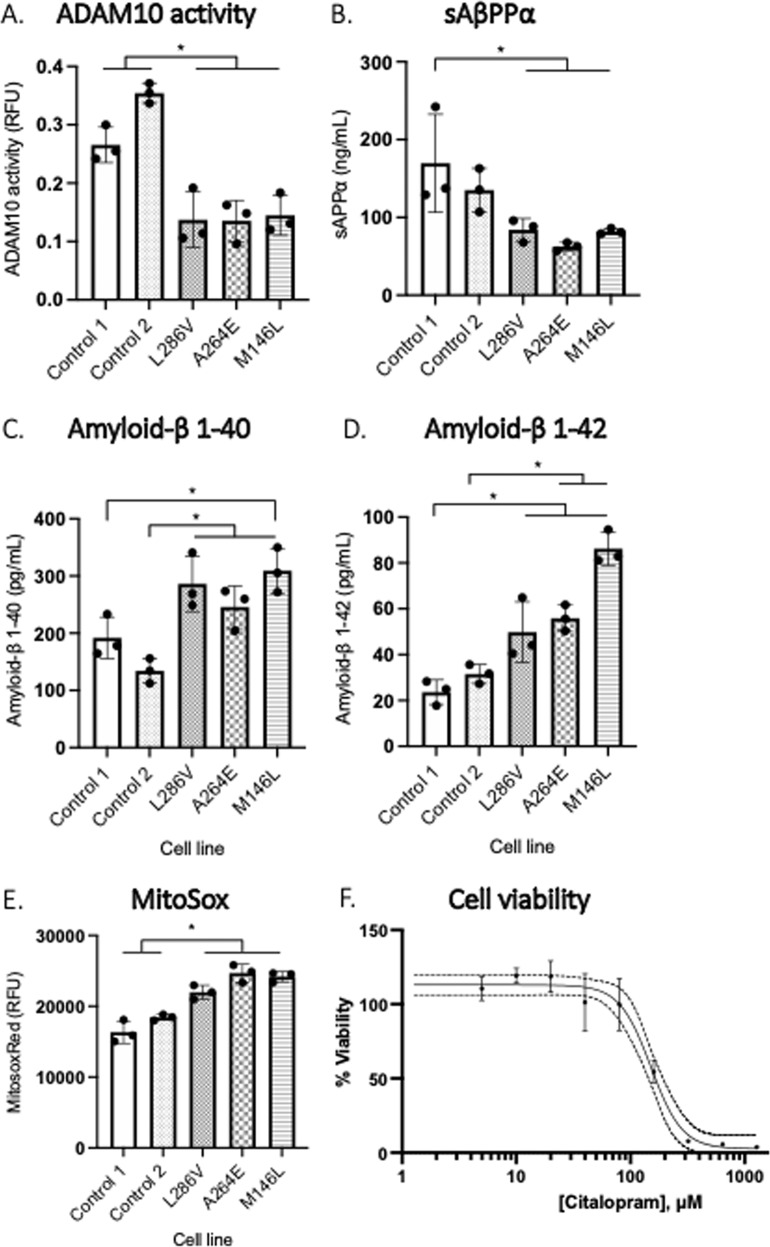
Graphs showing comparison between control and PSEN1 cell lines (d45) for baseline (untreated) measures. **A** ADAM1O activity (RFU) measured via FRET assay and **B** sAβPP-a (ng/mL) in media collected after 48 hrs indicate levels of non-amyloidogenic AβPP processing (*n* = 3). Media concentration between control and PSEN1 carrying cells (d45) of **C** Aβ1:40 (pg/mL) and **D** Aβ1:42 (pg/mL) analysed via ELISA (*n* = 3). **E** superoxide generation (MitoSox Red, RFU) were quantified in live cells (*n* = 3, d45). **F** Percentage viability after treatment with Citalopram (µM) measured via MTT assay in control cells (d45, *n* = 3). For multiple comparisons analysis with Bonferroni corrections, data normality (*p* > 0.05) and S.D. Variation (*p* < 0.05) were confirmed. *Significantly different to matched Cell Line (*p* < 0.05).

ICC staining was used to characterise the neuronal maturation of hNPCs and showed positive identification of MAP2 and synaptophysin after 45-days of culture (Fig. 1A). Further, AβPP, ADAM10 (Fig. 1Aii), and SERT receptors (Fig. 1Aiii.) were identified. Next, the functional response of neurons to exposure to 5HT as a receptor agonist was quantified by measuring calcium transients (Fig. 1B, C). The area under the curve (Fig. 1D) was significantly higher during exposure to 5HT (9.49 ± 7.00) compared to baseline measurements (3.51 ± 1.34, *p* < 0.05) before decreasing with washout of 5HT (6.36 ± 3.95) (*n* = 3). This was also seen in peak fluorescence intensity (RFU), with 5HT treatment (19.75 ± 15.66) resulting in a significant elevation above baseline (4.55 ± 2.31, *p* < 0.05) and 5HT wash out (9.16 ± 7.11, *p* < 0.05). This demonstrated that the cell models were able to respond to the desired action of SSRIs. Baseline markers of AβPP processing and redox status in PSEN1 L286V, A264E, and M146L cells were analysed against control to investigate the effects of the PSEN1 mutation on neuronal characteristics. ADAM10 activity was significantly lower in PSEN1 cells (0.14 ± 0.03 RFU) compared to Controls (0.31 ± 0.05 RFU, *p* < 0.01) at baseline (Fig. 2A). This was matched by lower sAβPPα secretion in PSEN1 (76.22 ± 12.99 ng/mL) compared to controls (152.4 ± 47.44 ng/mL, *p* < 0.05), which is indicative of reduced non-amyloidogenic AβPP processing in cells carrying a PSEN1 mutation (Fig. 2B). Further, elevation of amyloidogenic AβPP processing was indicated in PSEN1 cells with increased generation of Aβ1-40 (280.3 ± 45.60 vs. 163.0 ± 41.46 ng/mL, *p* < 0.01) and Aβ1-42 (76.28 ± 36.33 vs 27.61 ± 6.087 ng/mL, *p* < 0.05) compared to control (Fig. 2C, D). The ratio of Aβ1-42:40 was not significantly different between control (0.18 ± 0.07) and PSEN1 L286V (0.27 ± 0.10, *p* = 0.10) cells. Aggregated Aβ oligomers were not significantly changed in PSEN1 cells (0.42 ± 0.21 ng/mL) compared to Control (0.24 ± 0.06 ng/mL, *p* = 0.07). Cellular redox status was quantified by measuring markers of superoxide production, lipid peroxidation (pg/mL) and protein (mg/mL) oxidative damage. Superoxide (^-^O_2)_ production, measured via the MitoSOX^TM^ probe, was significantly elevated in PSEN1 cells (17387 ± 1538, RFU) relative to control (23611 ± 1520, RFU *p* < 0.05) (Fig. 2E). There were no significant differences observed in protein carbonylation between control (13.96 ± 4.13, mg/mL) and PSEN1 cells (22.71 ± 11.16 mg/mL, *p* = 0.10). In contrast, 8-isoprostane as a marker of lipid peroxidation, was significantly elevated in PSEN1 cells (4.46 ± 1.44 pg/mL) compared to control (1.94 ± 1.25, pg/mL *p* < 0.05). Cytotoxicity of the SSRI, Citalopram, was then quantified by MTT assay (Fig. 2F). From the dose-response curve lower doses of Citalopram were well tolerated. The best fit model predicted a maximum viability response (EMAX) of 113.1% and a minimum viability of 2.559% between 0 and 1280 µM Citalopram. A 50% loss in viability (IC50) with Citalopram was at a concentration of 150.90 µM with a hill slope of -3.17 compared to 0 µM. One-way ANOVA, grouped analysis, showed a significant reduction in percentage cell viability with 320 µM Citalopram treatment (25.75 ± 30.26%) compared to 0 µM (100.00 ± 6.28%, *p* < *0.05*).

### Non-amyloidogenic AβPP processing is increased following Citalopram treatment in PSEN1 cells

**Fig. 3:**
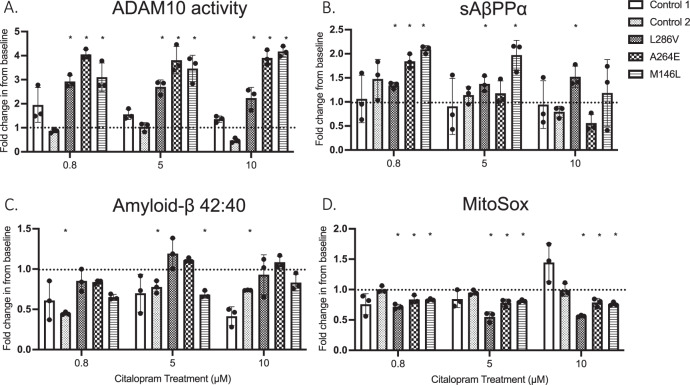
Bar graphs showing data for markers of AβPP processing and superoxide production following treatment with Citalopram (µM) for 44 days. Key indicates representative bars corresponding to cell lines, with each bar representing fold change from cell line baseline measurement (dotted line). **A** ADAM10 activity measured in live cells via FRET assay (RFU). **B**, **C** sAβpp-a (ng/mL) and Aβ42:40 (pg/mL) respectively, measured in spent (after 48 h) cell culture media. **D** Mitochondrial Superoxide generation (MitoSox Red, RFU) quantified in live cells. Replicates were collected are day 45 following 44 days of Citalopram treatment (*n* = 3). For multiple comparisons analysis with Bonferroni corrections, data normality (*p* > 0.05) and S.D. Variation (*p* < 0.05) were confirmed *Significantly different to Citalopram treatment (*p* < 0.05).

Both Control and PSEN1 cells were treated with Citalopram for 44 days at concentrations of 0 µM, 0.8 µM, 5 µM and 10 µM. ADAM10, responsible for α-cleavage of AβPP, showed a significant fold change (FC) increase in enzyme activity with Citalopram treatments in PSEN1 cells, 0.8 µM (3.36 ± 0.62 FC, *p* < 0.05), 5 µM (3.32 ± 0.65) and 10 µM (3.44 ± 0.96), relative to control. ADAM10 activity was not changed in control cells (Fig. 3A). The cleavage product sAβPPα was not changed in Control cells but was significantly increased in PSEN1 cells following treatment with 0.8 µM Citalopram (2.150 ± 0.8566, *p* < 0.05), relative to control cells. There was no significant change with either 5 µM (1.508 ± 0.4178) or 10 µM (1.089 ± 0.5649) treatment (Fig. 3B).

### Treatment with Citalopram has little effect on Aβ production in Control cells and PSEN1 cells

Aβ generation and aggregation was measured in 48 hr spent media. No significant changes were evident in Aβ1-40 or Aβ1-42 in either cell line. The ratio of Aβ1-42:40 was significantly decreased in control cells at 0.8 µM (0.53 ± 0.18 AU, *p* < 0.05), and 10 µM (0.58 ± 0.19 AU, *p* < 0.05), this effect was not seen in PSEN1 cells (Fig. 3C). Aggregation of Aβ peptide into oligomers was not altered in control or PSEN1 cells with Citalopram treatment.

### Citalopram treatment effects redox balance differentially in both control and PSEN1 cells

Fold change in ^-^O_2_ production was not significantly changed in Control cells. In PSEN1 cells, in ^-^O_2_ production was significantly lowered following treatment with 0.8 µM (0.79 ± 0.07), 5 µM (0.71 ± 0.13) and 10 µM (0.70 ± 0.1127) Citalopram relative to 0 µM (*p* < 0.05) (Fig. 3D). There was no change in either protein carbonyl formation or lipid peroxidation in either cell line following Citalopram treatments.

## Discussion

This study investigated the effect of Citalopram treatment on AβPP processing and redox balance using a hNPC-derived neuronal models carrying PSEN1 L286V, A264E and M146L mutations. In similar models, these mutations have been previously shown to reflect key the biochemical changes that are seen in AD [8, 9]. The data presented suggests that Citalopram treatment had little effect on the generation of Aβ in fAD PSEN1 cell models but did significantly increase ADAM10 activity and sAβPPα secretion. This suggests Citalopram treatment caused an increase in non-amyloidogenic AβPP processing. Citalopram treatment also reduced superoxide generation in PSEN1 cell models, which indicates that it can offer some protection against oxidative stress. Thus, SSRIs may protect against the development of AD pathology early in the disease process.

The application of cell reprogramming techniques to develop models of disease that closely resemble human biochemistry has been valuable, especially in conditions with a known genetic onset [14]. In this study, Aβ1-40, Aβ1-42 and free radical production were increased in neurons carrying the PSEN1 mutation. These data agree with previous research that have shown altered Aβ generation in PSEN1 carrying cortical cell types [8, 9, 15, 16]. In this study, we also identified a reduction in ADAM10 enzyme activity in PSEN1 L286V cells. Strategies to increase ADAM10 activity have been proposed as a target for the treatment of AD [17].

The therapeutic effects of SSRIs have been previously shown to stimulate non-amyloidogenic AβPP processing [7]. In the present study, Citalopram had little effect on ADAM10 activity or the liberation of sAβPPα in control cells. However, in PSEN1 L286V cells, which had reduced baseline α-cleavage of AβPP, both ADAM10 activity and sAβPPα liberation were significantly elevated by Citalopram treatment, presenting evidence that Citalopram may act to restore physiologically ‘normal’ levels non-amyloidogenic AβPP processing. It is noteworthy that ADAM10 activity was not directly related to sAβPPα levels. This is likely due to the multiple targets of ADAM10 but more research is required to better understand the regulation of ADAM10 enzymatic control [18] by SSRIs. Future research into this may benefit from the use of isogenic control lines to investigate ADAM10 regulation, as ADAM10 activity can be altered by a number of pathways. However, in this study it is clear that citalopram was able to increase ADAM10 activity in PSEN1 cells when compared to no treatment.

Data presented herein showed that Citalopram treatment had little effect on amyloidogenic AβPP processing. In control cells a decrease in the Aβ1-42:40 ratio was evident, which has been linked to AD pathology due to the tendancy of longer Aβ peptides more readily self-aggregating [19]. However, in ‘healthy’ control cells, no change in Aβ aggregation was detected. This may be explained by lower levels of aggregation in Aβ in ‘healthy’ cells and so AβPP processing is at physiologically normal levels at baseline. In contrast, Citalopram treatment had no effect on Aβ generation in PSEN1 cells. To add to this data, it would be interesting to examine whether the clearance of Aβ Is altered by SSRI treatment as this is known to be reduced in late-onset AD [20], however, the current study is unable to answer this question as Aβ degrading enzymes were not measured. Equally the lack of a blood brain barrier structure in this model is a consideration for utility of iPSC derived cell models in drug delivery research.

Disrupted redox balance has been proposed as one of the earliest events in AD pathology [21], and the results presented herein suggest altered radical production was evident in PSEN1 cells, however, this was not translated into modified end products associated with oxidative distress. Further, in PSEN1 cells, Citalopram reduced ^-^O_2_ production at all concentrations tested. This suggests that Citalopram may confer protection against oxidative stress, although whether this reduction in radical production would alter cellular redox status is not known. Protein oxidation has been identified in the brains of people with AD [22, 23] yet, this was not supported in our models. This may be explained by the nature of the global protein carbonylation assay used, whereby specific protein functions are compromised by oxidation. Instead, future research looking at specific protein modifications may be more insightful [24].

There are some limitations to this research. This study has focussed on neurons, and thus cannot present information on the effect of Citalopram specifically on critical glial cell types that are known to regulate key functions in the brain. For example, astrocytes are known to support neuronal function and can protect against neuronal damage and themselves can be dysfunctional in AD [25, 26]. As methodology develops, studies will inevitably work more with mixed cell models to understand the interactions between cell types and the effect that this may have on responses to treatment.

In conclusion, this research highlights hNPC-derived neuronal models as a potential tool for screening therapeutic treatments for AD. Evidence was provided to show that PSEN1 carrying cells have perturbed features linked to AD pathology. Treatment with Citalopram appears to have little effect on Aβ generation in PSEN1 cells, but our findings suggest that treatment can significantly increase non-amyloidogenic AβPP processing and reduce oxidative stress. These changes may explain why SSRIs appear most effective in the prodromal period of the disease progression, as opposed to reducing established AD pathology. Further investigation of specific pathways conferring the beneficial effects of Citalopram treatment are warranted.
